# A Rare Case of Ixazomib-Induced Cutaneous Necrotizing Vasculitis in a Patient with Relapsed Myeloma

**DOI:** 10.1155/2019/6061484

**Published:** 2019-10-20

**Authors:** Heather Katz, Mina Shenouda, Deena Dahshan, George Sonnier, Yehuda Lebowicz

**Affiliations:** ^1^Department of Hematology/Oncology, Donald and Barbara Zucker School of Medicine at Hofstra/Northwell, 500 Hofstra Blvd, Hempstead, NY 11549, USA; ^2^Department of Hematology/Oncology, Joan C. Edwards School of Medicine, Marshall University, 1600 Medical Center Dr., Huntington, WV 25701, USA; ^3^Joan C. Edwards School of Medicine, Marshall University, 1600 Medical Center Dr., Huntington, WV 25701, USA; ^4^Department of Dermatology, University of Louisville School of Medicine, Louisville, KY 40203, USA; ^5^Department of Hematology/Oncology, UPMC Hillman Cancer Center, 1030 Beaver Hollow Rd., Beaver, PA 15009, USA

## Abstract

Ixazomib is the only oral proteasome inhibitor used in relapsed/refractory myeloma. Cutaneous side effects due to ixazomib have been documented in the literature; however, cutaneous necrotizing vasculitis is extremely rare. We describe a case of a 74-year-old man with relapsed multiple myeloma who was started on ixazomib, lenalidomide, and dexamethasone. He developed several skin lesions that were biopsied and revealed cutaneous necrotizing vasculitis. Ixazomib was held with resolution of the vasculitic lesions and restarted with dexamethasone to 20 mg on the day of treatment and 20 mg dose the day after treatment.

## 1. Introduction

There are several approved treatments for relapsed/refractory multiple myeloma; however, only one completely oral triplet regimen is approved. The combination of ixazomib, a proteasome inhibitor, lenalidomide, an immunomodulatory medication, and dexamethasone, a steroid, IRd, is the only triplet regimen in which all of the medications can be taken by mouth providing convenience to the patient as they do not need to come to the clinic as frequently for treatment. Typically, IRd is well tolerated with common toxicities including gastrointestinal issues, rash, thrombocytopenia, peripheral edema, and peripheral neuropathy. Cutaneous adverse events should be monitored with ixazomib and rash, and urticaria and dry skin have been discussed in the literature. Necrotizing cutaneous vasculitis due to treatment from ixazomib is extremely rare and has only been reported once in the literature. We report a case of ixazomib-induced necrotizing cutaneous vasculitis in a 74-year-old-male treated with ixazomib for relapsed myeloma that resolved by holding the medication. He was restarted on ixazomib plus steroids with no recurrence of cutaneous vasculitis and no complications of increased steroid dose.

## 2. Case Report

**Figure 1:**
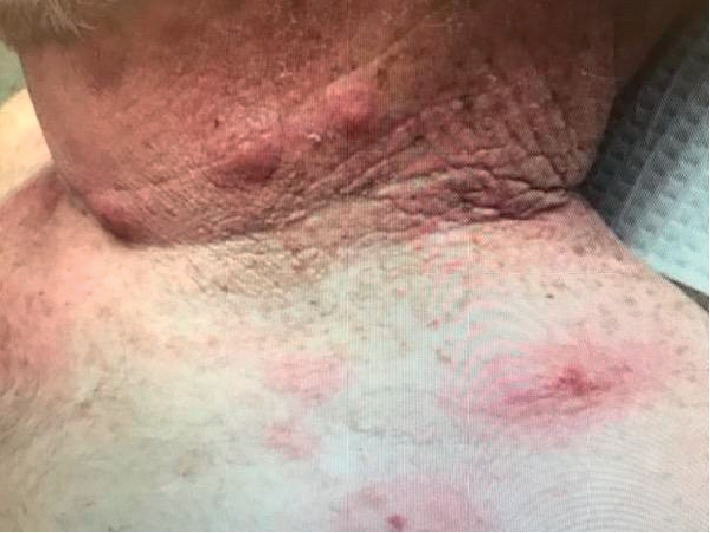
Multiple small lesions on his neck and chest.

**Figure 2:**
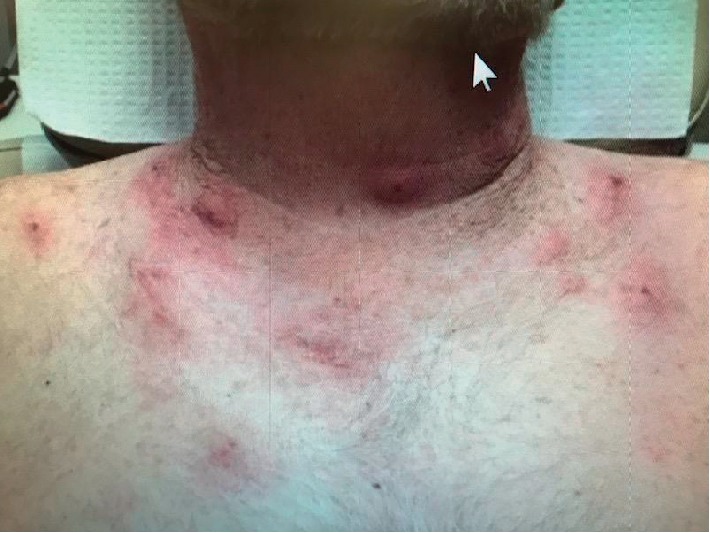
Multiple small lesions on neck and chest.

A 74-year-old-male with a past medical history of bronchitis, carpal tunnel, COPD, depression, gout, and hypertension was initially diagnosed with IgG Kappa smoldering myeloma in 2006. He was monitored with close surveillance until 2014 when he developed back pain. MRI of his spine showed a T-9 vertebral fracture which was biopsied. Final pathology was consistent with plasma cell neoplasm. In June 2014, he had a bone marrow biopsy which revealed 21% plasma cells. Myeloma FISH analysis showed monosomy 13 and gain of chromosomes 7, 9, and 15. Cytogenetics was normal. He received palliative radiation to T-9 and was started on lenalidomide 25 mg, days 1–21 of a 28-day cycle and dexamethasone 20 mg weekly. He was also started on zolendronic acid 4 mg IV every 3 months. Lenalidomide and dexamethasone were discontinued after 18 months due to patient preference. In February 2018, a PET-CT scan was performed and showed bilateral rib uptake associated with healing and nondisplaced fractures as well as left femur greater trochanter uptake secondary to a nondisplaced fracture. Repeat bone marrow biopsy in March 2018 showed 30% involvement with plasma cells. He was started on lenalidomide, bortezomib, and dexamethasone (RVd) without side effects. About 6 months after starting RVd, due to difficulty getting to the clinic, he was started on oral triplet therapy including lenalidomide 25 mg days 1–21, ixazomib 4 mg days 1, 8, and 15, and dexamethasone 20 mg days 1, 8, 15, and 22. After one week of being on this regimen, he developed multiple small lesions on his neck and chest (Figures 1 and 2).

**Figure 3:**
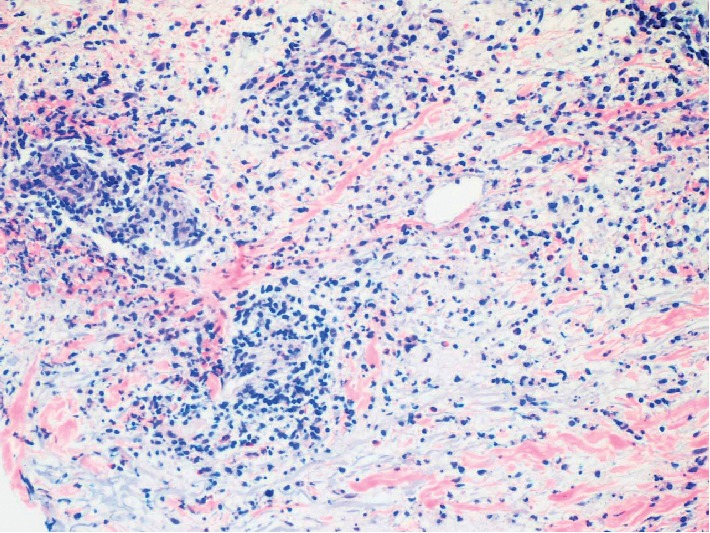
Prominent neutrophilic component of intense mixed dermal inflammation and vascular destruction from vasculitis (200x).

The patient was told to hold the ixazomib and presented to the dermatologist for a biopsy. Biopsy revealed intense dermal and pannicular infiltrate that is neutrophil rich and demonstrates overlapping features between Sweet's syndrome and the necrotizing vasculitis process (Figure 3).

**Figure 4:**
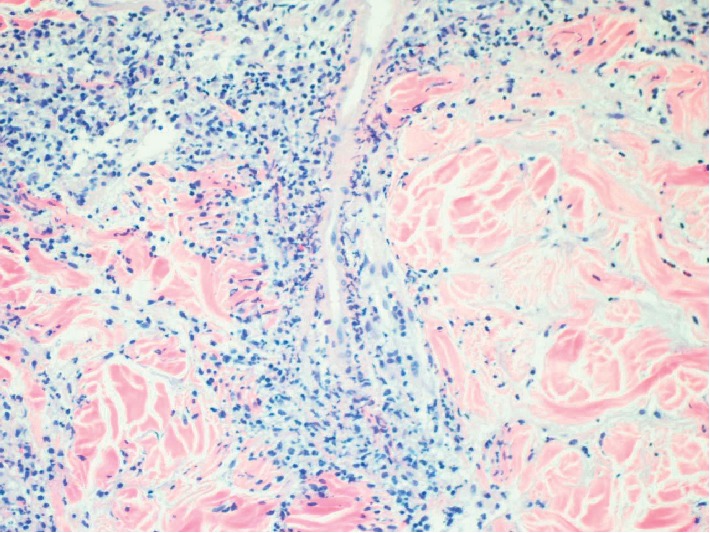
Deep dermal inflammation with leukocytoclastic vasculitis (200x).

Vascular destruction was seen confirming the concept of leukocytoclastic vasculitis (Figure 4).

Ixazomib was held and the lesions resolved completely. After complete resolution of the lesions, he was restarted on ixazomib with decadron 20 mg on the day of and 20 mg day after Ixazomib treatment and has not had further skin lesions. Workup for systemic vasculitis was also negative. Three-month follow-up revealed no further cutaneous manifestations and no other complications due to increased steroid dose.

## 3. Discussion

Multiple myeloma is a clonal plasma cell hematologic malignancy [1]. Despite initial treatment, patients with multiple myeloma often relapse or become refractory to treatment requiring a change in treatment [1]. The current preferred treatment regimens for patients with initial relapse receiving at least one prior therapy include proteasome inhibitors, immunomodulatory drugs, steroids, and monoclonal antibodies, commonly administered as a combination of 2 or 3 drugs [1]. Although there are several combinations approved in the setting of relapsed/refractory myeloma, the only orally available regimen for patients is the combination of ixazomib, lenalidomide, and dexamethasone (IRd). This oral regimen offers convenience to patients and clinicians as patients only need to return to clinic monthly for clinical assessment and review of laboratory data.

Ixazomib, or Ninlaro, is the first and only FDA-approved oral proteasome inhibitor. It is used in combination with lenalidomide and dexamethasone for multiple myeloma patients who received at least one prior treatment [2]. Ixazomib is an oral proteasome inhibitor alternative that may be preferred to bortezomib or carfilzomib based on its side effect profile, especially in patients with significant pre-existing neuropathy or relapse to bortezomib; however, efficacy of ixazomib compared with carfilzomib or bortezomib is unknown as trials approving ixazomib were compared regimens without a proteasome inhibitor [1]. Moreau et al. [3] and Hou et al. [4] discussed the safety and efficacy of ixazomib in patients with refractory or recurring myeloma, and further studies are evaluating the role of ixazomib as a maintenance therapy for multiple myeloma [5]. The combination of ixazomib, lenalidomide, and dexamethasone demonstrated significantly longer progression-free survival rates when compared with placebo, lenalidomide, and dexamethasone [3], as well as overall survival, which favored the IRd group [4]. Ixazomib with lenalidomide and dexamethasone treatment also revealed a manageable tolerability profile, with data reporting gastrointestinal issues, rash, thrombocytopenia, peripheral edema, and peripheral neuropathy as common toxicities [3–6]. Cutaneous adverse events of rash, dry skin, and urticaria have been identified in association with ixazomib. In the phase III trial that led to ixazomib's approval for relapsed myeloma, 36% of the ixazomib group experienced rash (grade 1 and 2) and 21% resolved without intervention compared to 23% in the placebo group with 12% resolving without intervention [3]. Cutaneous necrotizing vasculitis is extremely rare, and its pathogenesis is unknown [7, 8].

Guidelines for ixazomib-induced cutaneous necrotizing vasculitis are currently ambiguous, and little characterization is available as it is not a commonly reported adverse event [1, 7, 8]. Recognizing cutaneous toxicities secondary to myeloma therapies is critical for early intervention [8]. Vasculitic skin lesions may manifest due to vasculitis also involving internal organs, requiring systemic workup to rule out other possible causes, making diagnosing cutaneous vasculitis a diagnosis of exclusion [9]. A single case report of cutaneous necrotizing vasculitis secondary to ixazomib documents two cases of ixazomib-induced vasculitis in a myeloma patient and a patient with Waldenstrom's macroglobulinemia. Both patients had negative systemic vasculitis workup and presented with rashes involving plaques with a single central purpuric macula. Skin biopsies demonstrated interface dermatitis, lymphocytic vasculitis, and mixed, acute, and chronic granulomatous necrotizing vasculitis involving small- and medium-sized vessels with a lot of eosinophils. Both patients received treatment for their respective diseases; however, dose reductions and low dose steroids were needed [8].

Eruption and spreading of the rash can be managed by administering a corticosteroid on nontreatment days, holding treatment for a week and reducing ixazomib dosage [8]. Topical steroids will only provide symptomatic relief, and in trials with bortezomib-induced cutaneous vasculitis, recurrence is prevented by premedication before treatments [10].

It is important for clinicians to recognize that cutaneous vasculitis with other myeloma treatments other than ixazomib exist, especially as treatment plans continue to evolve and often include multiple medications that can cause cutaneous manifestations. Both lenolidamide and bortezimib, which our patient was on prior to ixazomib for approximately 6 months without issue, can also cause cutaneous vasculitis [11, 12] Vasculitis due to bortezomib typically appear after 2 cycles although they can erupt within cycles 1–4 [11] Although possibly induced by one of the other medications he was previously exposed to, our patient's cutaneous vasculitic presentation was most likely due to ixazomib as that was the only new medication prior to his skin manifestations.

## 4. Conclusions

Close monitoring for skin reactions is important following treatment with ixazomib. Recognizing and identifying cutaneous adverse events, especially in rare cases of necrotizing vasculitis, allow for early intervention and management while allowing for a continued effective treatment for relapsed myeloma. Cutaneous necrotizing vasculitis should be recognized and understood by clinicians and considered as a differential diagnosis when following relapse/refractory myeloma on ixazomib.
